# In vitro organo-protective effect of bark extracts from *Syzygium guineense var macrocarpum* against ferric-nitrilotriacetate-induced stress in wistar rats homogenates

**DOI:** 10.1186/s12906-016-1263-1

**Published:** 2016-08-26

**Authors:** Francine Nzufo Tankeu, Constant Anatole Pieme, Cabral Prosper Biapa Nya, Romain Jacques Njimou, Bruno Moukette Moukette, Angelo Chianese, Jeanne Yonkeu Ngogang

**Affiliations:** 1 Department of Biochemistry and Physiological Sciences; Faculty of Medicine and Biomedical Sciences, Laboratory of Biochemistry, University of Yaoundé I, PO Box 1364, Yaounde, Cameroon; 2 Laboratory of Medicinal plant Biochemistry, Food Science and Nutrition, Department of Biochemistry, Faculty of Science, University of Dschang, PO Box 67, Dschang, Cameroon; 3 Department of Chemical Materials Environmental Engineering, University of Rome “La Sapienza”, Via Eudossiana No. 18, Piazzale Aldo Moro 5, 00185 Rome, Italy

**Keywords:** *Syzygium guineense var macrocarpum*, SOD, Peroxidase, Malondialdehyde homogenates, Glutathione

## Abstract

**Background:**

Overconsumption of oxygen in mammalian cells often lead to the production of reactive oxygen species (ROS) resulting from different mechanisms. Escape of scavenging enzymes/components or nutritional failure are the most important origins. Plant-derived molecules may protect biological molecules either by quenching free radicals, delaying or preventing the ROS formation or by restoring antioxidant enzymes activities. The present study assessed the antioxidant, phenolic profile and protective effect of barks extracts of *Syzyguim guineense var macrocarpum* against ferric nitriloacetate-induced stress in the liver, heart kidney and brain tissues of wistar rat homogenates.

**Methods:**

Three extracts (aqueous, ethanol and aqueous-ethanol) from the barks of *S. guineense var macrocarpum* were used in this study. The spectrophotometric standardized methods were used to determine the free radical scavenging and antioxidant potential of the extracts. The protective properties of these plant extracts were also investigated as well as the quantification of secondary metabolites content (total phenolic, flavonoids and flavonols content). The HPLC method helped for characterizing phenolic compounds present in these extracts.

**Results and Discussion:**

All the extracts exhibited a free radical scavenging potential in a concentration dependent manner which varied from 15.18 ± 0.80 to 97.15 ± 0.71 % depending to the type of extract and the method used. The ethanol extract had the higher phenolic content (432.85 mg QE/g extract), including total flavonoids (961.66 mg QE/g extract) and flavonols content (25.12 mg QE/g extract) and higher total antioxidant capacity. Among the phenolic compounds present in the extracts, the HLPC profile revealed the presence of syringic acid and apigenin in all the extracts. The extracts demonstrated their protective effect mostly in liver and brain homogenates by delaying or preventing lipid peroxidation, restoring enzymatic activities and enhancing glutathione levels.

**Conclusion:**

The overall results demonstrated that the extracts exhibited significant antioxidant and protective effects in liver and brain liver homogenates.

## Background

Free radicals are produced from various physiological mechanisms owing to their significant biological roles in a myriad of signaling pathways at a reasonable dose [1, 2]. They are involved in defense mechanisms against pathogenic microorganisms and/or cancer cells and detoxification of harmful molecules [3]. Besides, reactive oxygen species (ROS) play vital roles in cells including stimulation of the signal transduction pathways, cell cycle regulation enzyme activation, and protein modification [4, 5]. In normal cells, free radicals are continuously produced in lower concentrations and can be neutralized by endogenous antioxidant species. Indeed, human body is endowed with protective mechanisms against deleterious effects of free radicals including both enzymatic and non enzymatic antioxidants [6]. However, when the naturally-occurring equilibrium between anti- and pro-oxidant agents is lost, higher ROS levels bypass the antioxidant function leading to a biochemical/physiological state described as oxidative stress [7, 8]. Oxidative stress refers to a state of imbalance between the production of free radicals and/reactive metabolites and removal of the latter by antioxidants [9]. Several factors are known to be responsible of this state. Among them, tobacco, alcohol, UV rays, medications, toxic metals, herbicides, nutritional failure or genetic defects responsible of non/poor expression of a gene encoding a protein or an antioxidant enzyme protein involved in the synthesis of an antioxidant could be listed [10]. Besides those exogenous factors, there are also endogenous sources mainly intracellular metabolism involving the mitochondrial respiratory chain, the defective autophagy of mitochondria and defective metabolism of redox metals [4, 11]. Oxidative stress can cause tremendous harmful effects including alteration of the protein structures and hence loss of vital functions. Evidence has indicated that oxidative stress is a potentiating factor of the onset of a broad range of diseases [12–14]. Among these, hemolytic anemia and β-hemoglobinopathies (sickle cell anemia and thalassemia), cancer, atherosclerosis and, Alhzeimer’s disease are believed to be aggravated by ROS [15–17]. These deleterious effects are counteracted by natural antioxidants [18, 19].

Numerous research results demonstrated that natural antioxidant molecules are anticarcinogenic anti-angiogenic and potent inhibitors of sickle cell haemoglobin polymerization [18, 20–22]. Phenolics-enriched plant extracts exhibited strong antioxidant and beneficial effect against chemically-induced stress in vitro [23–25] and in vivo [26, 27]. This justifies the upsurge of interest that naturally-occurring antioxidants from spices, vegetables, and herbs has been gaining [16, 23, 25, 28].

*Syzygium guineense* (Wild) DC. is a leafy forest tree of the Myrtaceae family, found in many parts of Africa both wild and domesticated which comprises three varieties. It is used in African traditional medicine to treat epilepsy, stomach-ache, diarrhoea, malaria, coughs, broken bones, wounds, asthma, sore throat, intercostal pain and as a tonic. The powdered bark is used as an antispasmodic and purgative [29]. The antibacterial properties of the aqueous extract of *S. guineense* have been demonstrated on different strains of bacteria responsible for diarrhea [30]. Ethanol extracts of the stem bark of *S. guineense* showed molluscicidal activities and cardioprotective properties, mainly due to the reduction of blood pressure [31]. Antibacterial activity of triterpenes isolated from *S. guineense* has been demonstrated [32]. Other biological properties such as anti-inflammatory, analgesic and immunological activities of different part of *S. guineense* have been reported [33]. The chemical composition of essential oil from *S. guineense* was also investigated [34]. A recent study demonstrated that leaves, stem bark and roots of *S. guineense* have antioxidant properties and are rich in polyphenols [23]. Almost all these biological properties are about the *guineense* variety. Up-to-date, no study investigating either the in vitro antioxidant activity or the protective effects of extracts of the *macrocarpum* variety has been carried out. Hence, this study attempted to investigate the in vitro free radical scavenging potential, antioxidant activity and the protective effect of *S. guineense var macrocarpum* barks extracts against ferric nitiloacetate-induced stress in the liver, heart and kidney and brain tissues of Wistar rats homogenates as well as their polyphenolic profile.

## Methods

### Plant material

Barks of *Syzygium guineense* var. *macrocarpum* were harvested in the surrounding islands of the *Sanaga* River (Centre region- Cameroon) in November 2014 and identified at the National Herbarium of Cameroon under the reference number 49885 *HNC*.

### Preparation of plant extracts

The harvested samples were cleaned, dried at room temperature and crushed. The powdered samples obtained were soaked separately in water, 95° ethanol, and the mixture water: ethanol (30:70;v/v) at pH = 3 for 48 h respectively. The mixtures were then filtered using Buchner funnel and Whatman N° 1 filter paper, concentrated under rotary evaporator. The aqueous and water:ethanol extracts were lyophilized while the ethanol extract was dried in an oven at 50 °C to obtain the crude extracts.

The resulting crude extracts were labelled as follows: SGFH_2_O: *Syzygium guineense var macrocarpum* aqueous extract (barks); SGFEtOH: *Syzygium guineense var macrocarpum* ethanolic extract (barks); SGF H_2_O/EtOH: *Syzygium guineense var macrocarpum* aqueous-ethanolic extract (barks). The crude extracts were stored at 4 °C until use. Before assaying each parameter, a stock solution of 1 mg/mL was prepared from which serial dilutions (0.025, 0.075, 0.150, 0.200 and 0.300 mg/mL) were prepared for the determination of the free radical scavenging activity. The phenolic metabolites content and antioxidant potential of different bark extracts were determined at 1 mg/mL.

### Determination of free radical scavenging and antioxidant properties

#### Determination of free radical scavenging activity

##### Scavenging activity of DPPH radical

This assay measures the free radical scavenging potential of each crude extract. The method described by [35] was used. Briefly, 1000 μL of a 0.1 mM DPPH ethanolic solution was added to 3000 μL of each diluted extract or Vitamin C used as standard. After 30 min of incubation in the darkness at room temperature the absorbance was measured at 517 nm against a blank.

### Scavenging effect of the ABTS^+^ radical

The radical scavenging capacity was measured by using ABTS^+^ solution radical cation. The assay was performed according to the method described by [36] with slight modifications. A stock solution of ABTS^+^ consisted of a 7.4 mM ABTS solution and 2.45 mM potassium persulfate solution in the ratio of 1:1. The mixture was allowed to react for 12 h at room temperature in the dark. A working solution was prepared by diluting 8 times the previous stock solution (20000 μL stock solution in 100000 μL volumetric flask, diluting it to the mark with ethanol) to get the absorbance of 0.7 ± 0.05 at 734 nm. After addition of 75 μL of extracts or vitamin C used as standard to 2000 μL of ABTS^+^ working solution, absorbance was measured at 734 nm after exactly 6 min.

The % inhibition for DPPH and ABTS assay was calculated according to the formula

$$ Scavening\kern0.5em  effect\kern0.5em \left(\%\right)=100\times \left({A}_o-{A}_s\right)/{A}_o $$

Where A_o_ is the absorbance of the blank; A_s_ is the absorbance of the sample

### Determination of antioxidant properties

#### Total antioxidant activity by Ferric Reducing Antioxidant Power assay (FRAP)

The FRAP assay was conducted following a previously described method [37] with slight modifications. The fresh FRAP reagent contained: acetate buffer (300 mM pH 3,6), 2,4, 6- Tri (2-pyridyl)-s-triazin (TPTZ) (10 mM) and FeCl_3_ · 6H_2_O (50 mM) in a 5:1:1 proportion respectively. FRAP reagent (2000 μL) was mixed to 75 μL of each tested extract and stored for 12 min. The activity of Vitamin C was used to plot the standard curve. The absorbance was read at 593 nm and results expressed as equivalent vitamin C/g of dried extract (mg eq Vit C/g DE).

#### Phosphomolybdenum antioxidant assay

The total antioxidant activity of extracts was evaluated by green phosphomolybdenum complex according to the method described by [38]. Phosphomolybenum reagent was prepared by mixing 0.6 M sulphuric acid, 28 mM sodium phosphate and 4 mM ammonium molybdate in 1:1:1 proportions. Phosphomolybdenum reagent (1000 μL) was introduced in test tubes. After addition of 10 μL of each extract sample, the mixture was homogeinized and the tubes incubated in a dry thermal bath at 95 °C for 90 min. Thereafter, tubes were cooled down and the absorbance of the mixture measured at 695 nm against a blank. The vitamin C was used as the standard and a calibration curve in the range of 0 – 0.3 mg/mL was prepared and BHT was used for the comparison. The reducing capacity of samples was expressed as mg of vitamin C equivalents/g of dried extract (mg vitC eq/g extract).

### Determination of total phenol content

Total phenol content of the spice extract was determined using Folin-ciocalteu method [39]. This method is based on the reduction of phosphotungstate-phosphomolybdate reagent in alkaline medium. In different test tubes, 200 μL of 1 mg/ml of sample were introduced. Then, 800 μL of 10 fold diluted Folin reagent and 2000 μL of sodium carbonate solution (7.5 %) were added. After stirring, the mixture was kept away from light for 2 h and the absorbance was measured at 765 nm. The phenolic content was determined from a quercetin standard curve. A concentration range from 0 to 0.3 mg/mL of quercetin was prepared and allowed to determine the total polyphenol content expressed in mg equivalents of quercetin/g of extract (mg QE/g extract).

### Determination of total flavonoid content

Total flavonoid content was determined using a well described method [40]. Briefly, 100 μL of extract were added to 300 μL of distilled water and 30 μL of NaNO_2_ (5 %). After 5 min of incubation at 25 °C, 30 μL of AlCl_3_ (10 %) were added. After further 5 min, the reaction mixture was treated with 200 μL of 1 mM NaOH and the reaction mixture diluted to 1000 μL with distilled water. Quercetin served to draw the standard calibration curve in the range of 0–0.3 mg/mL and the absorbance was measured at 510 nm. The results were expressed as mg quercetin equivalents/g of dried extract (mg QE/g extract).

### Determination of total flavonol content

Total flavonols content in the plant extracts was determined according to the previously described technique [41] with slight modifications. In different test tubes, each extract (2000 μL) and standard solutions (2000 μL) were placed and then 2 % aluminum chloride (2000 μL), 50 g/L sodium acetate (3000 μL) were added and mixed well. The mixture was incubated at 20 °C for 2.5 h and absorbance was read at 440 nm. Total flavonols content was calculated as mg quercétine equivalent/g of extract using the equation based on the calibration curve and expressed as mg quercetin/g of dried extract (mgQE/g extract).

### Determination of the polyphenolic content by HPLC

High Performance Liquid Chromatography (HPLC) with UV detection is frequently used to separate and characterize phenolic compounds present in extracts. The polyphenolic profile was determined according to a previously described method [28]. The analysis was performed on an Agilent Technologies 1200 HPLC system fitted with a SUPELCOSIL LC-18 column (length 250 mm, diameter 4.6 mm, packaging size 5 mm). Samples were dissolved in pure water to reach the concentration (300 mg/10 mL) and centrifuged at 4706 rpm for 10 min. The obtained supernatant was filtered through a cellulose acetate membrane filter (0.20 μm or 0.45 μm, Schleicher & Schuell). 25 μL of filtrate were injected into the HPLC system and eluted as described below.

The column temperature was set at 20 °C. The mobile phase consisted of a mixture of an aqueous solution of acetic acid at 0.5 % by volume (“A”) and acetic nitrile (“B”). Elution was performed by following this protocol: At start and for the first 2 min of the run, 100 % of A. From 2 to 60 min after the run start, a linear composition ramp was used, targeting 40 % of A and 60 % of B.

The flow rate was set to 1 mL/min. Polyphenols were detected by a UV detector (280 nm). Beforehand, the retention times of the identified polyphenolic compounds of interest available were measured by using of single standard solutions at a concentration of 100000 mg/mL. The quantification of identified compound was based on the area under peak determined at 280 nm and expressed relative to each corresponding phenolic standard.

### Evaluation of organ protective effects of plant extracts

#### Preparation of different tissue homogenates

Normal albino wistar rats (10) were sacrified and the organs (liver, kidney, brain and heart) were isolated and weighed. Each homogenate was prepared by mixing 10 % (w/v) of each ground organ and phosphate buffer (pH 7, 0.1 M) followed by a centrifugation at 3000 rpm for 30 min. The study was approved by the Faculty of Medicine and Biomedical Sciences Ethical committee authorizing the use of animals.

#### Preparation of ferric-nitrilotriacetate solution

The oxidizing solution was prepared according to [42] Briefly,1.62 g and 7.64 g of FeCl_3_ and NTA were dissolved in 100000 μL of HCl 0,1 N to reach the concentrations of 200 mM and 400 mM respectively. The obtained solution was then mixed to a H_2_O_2_ 200 mM 1:1 (v/v) The oxidant solution was prepared immediately before utilization.

### Total protein content

The total protein content of the mixture of liver was measured according to the protein kit supplier methods (Human Kit-Hu102536, Boehringer, Ingelheim, Germany). This result was used to express the activities of the different enzymes per g of organs.

### In vitro lipid peroxidation assay

The capacity of the spice extract to inhibit the lipid peroxidation was evaluated according to a previously implemented method [43]. In brief, 580 μL of phosphate buffer (0,1 M; pH 7,4), 200 μL of spice extract and 200 μL of each homogenate were successively introduced in different test tubes. Lipid peroxidation was then initiated by adding 20 μL of oxidizing solution (0.1 M HCl, FeCl_3_ 200 mM, 400 mM NTA, 200 mM H_2_O_2_) in the mixture. The whole was thereafter placed in a water bath at 37 °C for 1 h. At the end of the incubation, 100 μL of this mixture was pipeted and placed in new tests tubes to which 1000 μL of MDA reagent (TCA (10 %) and 1 ml of TBA (0.67 %) were added to terminate the reaction. All the tubes were then heated again at 100 °C for 20 min and transferred to an ice bath to be cooled and centrifuged at 3000 rpm for 5 min. The optical density was measured at 535 nm and the concentration of MDA was calculated using the formula:

$$ \begin{array}{l}\mathrm{O}\mathrm{D} = \varepsilon \mathrm{C}\mathrm{l}\ \mathrm{and}\ \mathrm{expressed}\ \mathrm{in}\ \mathrm{n}\mathrm{M},\ \mathrm{where}\ \varepsilon\ \mathrm{molar}\ \mathrm{extinction}\ \mathrm{coefficient} = 1.56 \times 1{0}^5/\mathrm{M}/\mathrm{cm}\ \mathrm{and}\ \mathrm{l}=\mathrm{length}\ \mathrm{of}\\ {}\mathrm{the}\ \mathrm{tank}.\end{array} $$

### Superoxide dismutase (SOD) activity assay

An indirect method of inhibiting autooxidation of epinephrine to its adrenochrome was used to assay SOD activity in plant-treated homogenates [44]. An aliquot consisting in (580 μL PBS, 200 μL of each extract or standard, 200 μL of liver, kidney, kidney, heart homogenate) and 20 μL of inducing solution was introduced in different test tubes and the obtained mixture was then incubated at 37 °C for 1 h to obtain the test solutions. The latter (test solutions) will be used to investigate the other enzymatic parameters as well as non enzymatic ones. To 20 μL of each test solution (Fe^3+^- NTA induced homogenates treated with plant extract or standard), 150 μL were added to 500 μL of carbonate buffer (pH 10.2; 0, 3 M; pKa 10.3), 250 μL of an EDTA solution (0.6 mM); The obtained mixture was then homogenized and 150 μL of an epinephrine solution (4.5 mm) were added to initiate the reaction. Four other tubes were run in the same conditions to serve as normal, negative and positive controls. The extract was replaced respectively by distilled water, oxidant, Vit C and quercetin.

The optical density was read after 30 min and 120 min at 480 nm. The following equation allowed the calculation of the SOD activity:

$$ SOD\left( unit/ mg\  protein\right)= SOD\left( unit s/ mL/ min\right)/ protein\left( mg/ mL\right)\times df $$

Where df = dilution factor

The SOD activity was thereafter expressed as Unit/min/mg of protein (UI/mg Prot.)

### Catalase activity

The catalase activity of plant extracts on different homogenates was assessed according to a formerly described method [45] with some amendments. The above tests solutions (100 μL) were dispensed in test tubes containing 900 μL phosphate buffer (0.01 M, pH 7). After homogenization the reaction was started by the addition of 400 μL of a hydrogen peroxide solution (200 mM), and after 60 s, 2000 μL of an acetic acid-dichromate solution were added to stop the reaction. The mixture was boiled for 10 min and the absorbance was measured at 530 nm.

### Glutathione peroxydase activity

In different test tubes, 580 μL of PBS (0.1 M; pH 7.4), 200 μL of each plant extract or vit C and quercetin used as standards, 200 μL of each homogenate (liver, heart, kidney and brain) and 20 μL oxidizing solution (HCl 0.1 M, FeCl_3_ 200 mM, NTA 400 mM, H_2_O_2_ 200 mM) were introduced. The normal control, negative and positive controls were run simultaneously in the same conditions except that, the oxidizing solution was replaced respectively by distilled water for the normal control, the plant extract by the distilled water for the negative control and vit C and quercetin for the positive controls. The mixtures were thereafter incubated at 37 °C for 1 h. Then, 100 μL of each of these mixtures were dispensed in new test tubes containing 900 μL of PBS (0,01 M; pH 7). An aliquot of PBS 0,01 M, pH 6; pH 7 (320 μL), hydrogen peroxide 0.05 % (160 μL), and pyrogallol solution 0.05 % (320 μL) were added to distilled water (210 μL). 100 μL from the above mixture was added thereafter. The reaction was mixed and incubated for at least 10 min and the increase in absorbance at 420 nm was measured after 20 and 140 s using a spectrophotometer.

### Reduced glutathione assay

The previously described method of Ellman [46], was used to determine glutathione antioxidant capacity of plant extracts. An aliquot of PBS (580 μL), 200 μL of extract and 200 μL of each homogenate (liver, kidney, brain and heart) and 20 μL of inducing solution was introduced in different test tubes. The obtained mixture was then incubated at 37 °C for 1 h. The above test solutions (20 μL) and 3000 μL of Ellman reagent (phosphate buffer 0,1 M; pH 6,5; 2,2-dithio-5,5′-dibenzoïc acid) were introduced in new test tubes. Glutathione concentrations were expressed in micromoles/L and calculated using the following formula:

$$ \mathrm{O}\mathrm{D} = \varepsilon \mathrm{C}\mathrm{l}\ \mathrm{where}\ \varepsilon \mathrm{glutathione} = 13600\ \mathrm{and}\ \mathrm{l} = \mathrm{optical}\ \mathrm{path}. $$

### Statistical analysis

The results were presented as mean ± SD of triplicate assays. Analyses of data was conducted using one-way ANOVA (Analysis of variance) followed by Kruskal wallis test and Dunnett’s multiple test (SPSS program version 18.0 for Windows, IBM Corporation, New York, NY, USA). The Log probit was used to determinate the IC_50_. XLstat version 7 (Addinsoft, New York, NY, USA) was used to achieve the Spearman rho Correlation Analysis as well as the principal component analysis (PCA). The differences were considered as significant at *p* < 0.05.

## Results and Discussion

A wide range of methods have been described for the free radical trapping potential and antioxidant properties of plant-derived components. DPPH is one of the most used assays to assess the antioxidant potential of tremendous naturally food derived and plant extracts. Results of DPPH free radical scavenging potential of different plant extracts are summarized in Table 1A. The DPPH free radical scavenging potential increases with the plant extract concentration. Inhibitory potential expressed as percentages ranged from (59.52 ± 0.56 to 93.57 ± 1.68 %) for the aqueous extract which presented a significant and lowest inhibitory potential compared to the ethanol extract (94.40 ± 0.13 %). However, Vitamin C had the most potent inhibitory potential (98.31 ± 0.27 %). These results suggest that these extracts are rich in hydrogen atom and or electron donating-substances as phenolic derived compounds, glycosylated derived compounds and anthocyans capable of pairing with the unstable DPPH^**o**^ radical [47].

**Table 1:** Radical scavenging potential of different barks extracts of *Syzygium guineense var macrocapum*

	Sample Concentration (μg/mL)	SGF H_2_O	SGF EtOH	SGF H_2_O/EtOH	VIT C
A	25	59.52 ± 0.56^c^	66.27 ± 0.95^b^	55.21 ± 1.28^bc^	74.43 ± 1.79^e^
	75	69.74 ± 0.16^c^	78.83 ± 0.95^d^	67.95 ± 1.36^c^	85.83 ± 0.66^e^
	150	79.30 ± 0.68^c^	82.45 ± 1.25^c^	79.84 ± 0.37^c^	88.45 ± 0.62^d^
	200	84.45 ± 0.61^a^	86.24 ± 4.73^a^	82.74 ± 0.14^a^	93.18 ± 2.58^b^
	300	93.57 ± 1.68^b^	94.40 ± 0.13^b^	88.82 ± 1.78^c^	98.31 ± 0.27^d^
	IC_50_ (μg/mL)	2.585	2.321	2.692	2.01
B	25	19.95 ± 1.61^d^	15.18 ± 0.80^a^	23.19 ± 0.66^d^	24.00 ± 1.22^a^
	75	36.47 ± 1.11^c^	39.10 ± 0.93^c^	26.54 ± 1.80^e^	64.91 ± 3.46^b^
	150	78.34 ± 0.71^b^	53.01 ± 0.36^c^	34.56 ± 1.94^e^	90.81 ± 2.40^cde^
	200	91.35 ± 0.15^c^	67.56 ± 1.29^d^	38.26 ± 0.66^e^	91.81 ± 1.57^de^
	300	97.15 ± 0.71^b^	81.37 ± 0.94^c^	52.63 ± 0.71^d^	93.68 ± 1.16^e^
	IC_50_ (μg/mL)	2.299	2.922	5.119	1.632

*Values are expressed as mean ± SD,* In the same column the values designated different letters are significantly different at *p* < 0.05

A: DPPH radical scavenging potential of different plant extracts, B: ABTS radical scavenging potential of different plant extracts

*SGFH*_*2*_ *O/EtOH* syzygium guineense var macrocarpum (barks) aqueous-ethanol, *SGFEtOH* syzygium guineense var macrocarpum (barks) ethanolic, *SGFH*_*2*_ *O* syzygium guineense var macrocarpum (barks) aqueous, *Vit C* vitamin C

Besides the scavenging of free radicals, group I antioxidants also quench protons to suppress their reactivity. ABTS^+^ cation scavenging potential of plant extracts was also investigated (Table 1B). Conversely to the results obtained with DPPH^•^, the aqueous extract was more efficient to inhibit ABTS radical among the extracts and Vit C with a scavenging percentage of 97.15 ± 0.71 % demonstrated the highest inhibitory effects. The difference in activity found here between the different tested extracts can be due to the difference in number of hydroxyl groups present in molecules extracted by each solvent. Indeed, previous studies showed that the higher the number of free hydroxyl groups present in polyphenols, the higher their scavenging potential [48]. Moreover, the position of the hydroxyl group also influences the activity of the molecule [49, 50]. The 50 % inhibitory concentrations (IC_50_) for the DPPH and ABTS^+^ radicals are displayed respectively in (Table 1A and B). From these results the ethanolic extract exhibited the lowest IC_50_ values (1.944 μg/mL and 3.871 μg/μL) both for the DPPH^•^ and ABTS^+^ free radical scavenging efficiency.

The chelation of transition metals such as iron is a crucial in the prevention of radical generation which damage target biomolecules. Indeed, iron acts as catalyst in the Harber-Weiss reaction to generate the highly reactive hydroxyl radical. FRAP assay was used to investigate the capacity of plant extracts to act as preventive antioxidants. Results are displayed on Table 2. All the tested samples demonstrated a preventive antioxidant potential with different capacity depending of the tested extracts. However, the aqueous extract showed the significant best ferric reducing power compared to the other samples. In addition to the ferric reducing antioxidant power, the phosphomolybdenum antioxidant capacity of plant extracts investigated to better elucidate the antioxidant mechanism demonstrated that the best antioxidant capacity was exhibited by the aqueous ethanol extract. Moreover, the antioxidant capacity of this extract was twice higher than that of Butylated HydroxyToluene (BHT) used as the standard antioxidant.

**Table 2:** Phenolic metabolites content and antioxidant potential of different barks extracts of *Syzygium guineense var marcarpum*

		SGFH_2_O	SGFEtOH	SGFH_2_O/EtOH	
Polyphenol content	Total phenol content (mg QE/g Ext.)	432.38 ± 11.41^a^	352.85 ± 3.83^c^	236.19 ± 3.09^d^	
	Flavonoids (mg QE/g Ext.)	961.66 ± 7.63^a^	716.66 ± 23.62^db^	316.66 ± 12.58^e^	
	Flavonols (mgQE/g Ext.)	25.12 ± 5.12^a^	18.15 ± 0.97^b^	4.02 ± 1.04^c^	
Antioxidant potential	Phosphomolybdenum	113.88 ± 13.36^a^	278.88 ± 32.50^a^	226.66 ± 38.35^c^	BHT
	FRAP	209.13 ± 3.17^a^	278.88 ± 5.75^d^	141.72 ± 1.57^a^	122.22 ± 2.5^a^
	(Total Antoxidant Capacity)				

*Values are expressed as mean ± SD,* In the same column, the values designated different letters are significantly different at *p* < 0.05

*SGFH*_*2*_ *O/EtOH* syzygium guineense macrocarpum (barks) aqueous-ethanol, *SGFEtOH* syzygium guineense macrocarpum (barks) ethanol*, SGFH*_*2*_ *O* syzygium guineense macrocarpum (barks) aqueous, *mg QE/g Ext.* milligram quercetin equivalent/gram of extract

**Fig. 1:**
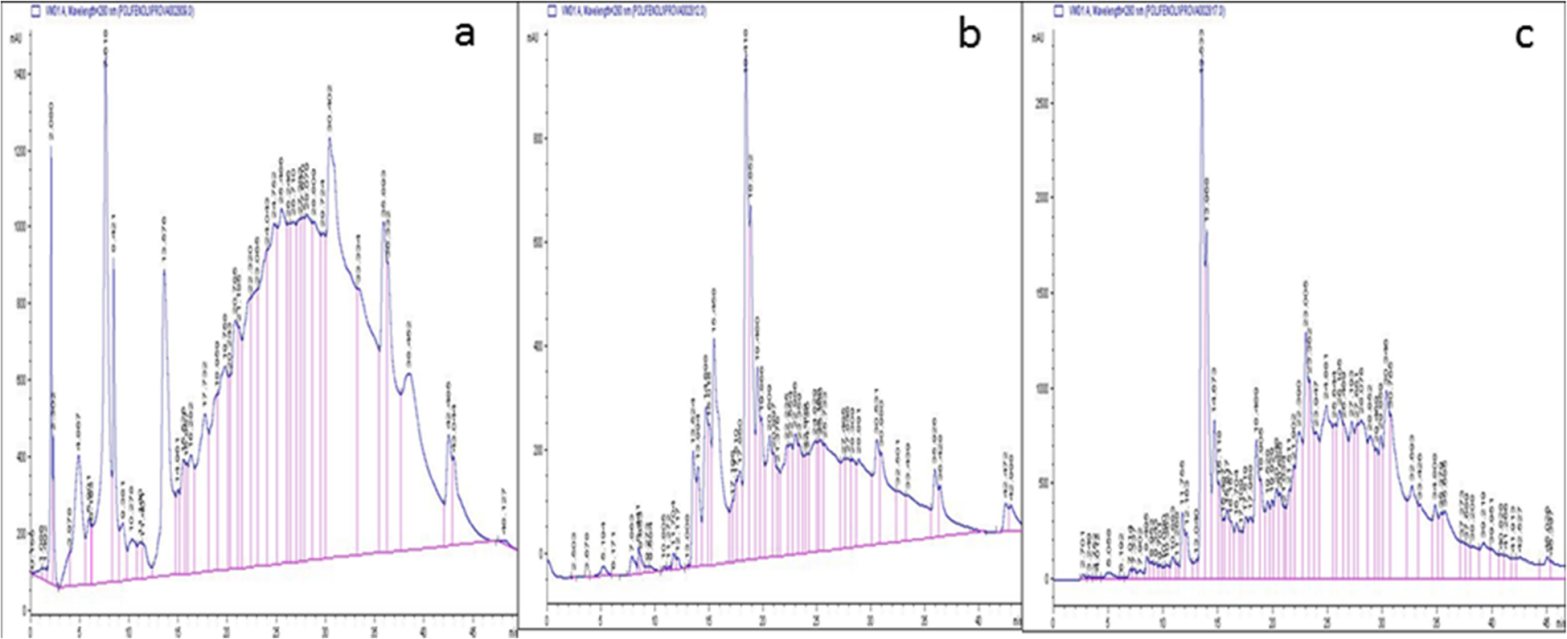
HPLC chromatogram profile of *Syzygium guineense var macrocarpum* (barks); **a**: aqueous extract; **b**: Ethanol extract; **c**: Aqueous ethanolic extract

Concerning the total phenol content, the ethanolic extract presented the highest content compared to the others. The same trend was observed with flavonoids and flavonols content (Table 2). These results highlighted the close relationship between the richness of plant extracts in polyphenols and their antioxidant capacity even though the antioxidant capacity depends on the mechanism involved. This suggestion is supported by the correlation coefficients values between total phenol content and FRAP assay (0.988), FRAP and flavonoids (1.00) and flavonol content (1.00) (Tables 3 and 4). The nature of the polyphenol and its concentration significantly affect the antioxidant activity of the extract. Our results from HPLC profile (Fig. 1 and Table 5) demonstrated a variety of polyphenol is different concentration which include phenolic acids (*p*-coumaric acid; syringic acid), flavonoids (catechin and caffeic acid) and other phenolic compounds.

**Table 3:** Correlation study between variables; (1) Correlation between scavenging potential and polyphenol metabolites; (2) correlation between enzymatic antioxidant and lipid peroxidation parameter from rat organs

Variables	DPPH	ABTS	Molyb	Frap	Flavo	Pheto	Flavonol
DPPH	**1**						
ABTS	0.381	**1**					
Molyb	0.000	**−0.810**	**1**				
Frap	0.195	**0.781**	0.390	**1**			
Flavo	0.195	**0.781**	0.390	**1.000**	**1**		
Pheto	0.193	**0.795**	0.410	**0.988**	**0.988**	**1**	
Flavonol	0.195	**0.781**	0.390	**1.000**	**1.000**	**0.988**	**1**

*Bivariate Spermann rho correlation.* Bold values are: significant coefficient, *p* = 0.05 (bilateral test)

*Molyb* phosphomolybdenum test; *Flavonols* flavonol assay; *Phetot* polyphenol assay; *Flavonoids* flavonoid assay; *ABTS* ABTS radical scavenging test; *DPPH* DPPH radical scavenging test; *Per* peroxidase; *SOD* superoxide dismustase; *CAT* catalase; *MDA* malonedialdehyde; *GLU* Glutathione; *L* liver; *H* heart; *K* kidney; *B* brain

**Table 4:** Correlation study between variables. Correlation between enzymatic antioxidant and lipid peroxidation parameters from rat organs

Variables	Per L	Per H	Per K	Per B	SOD L	SOD H	SOD K	SOD B	CAT L	CAT H	CAT K	CAT B	MDA L	MDAH	MDAK	MDAB	GLU L	GLU H	GLU K	GLU B
Per L	**1**																			
Per H	0.321	**1**																		
Per K	0.321	0.071	**1**																	
Per B	0.107	0.107	**0.643**	**1**																
SOD L	0.250	0.036	**0.786**	**0.643**	**1**															
SOD H	0.429	0.179	**0.750**	0.464	**0.964**	**1**														
SOD K	0.107	0.286	0.500	0.536	**0.893**	**−0.821**	**1**													
SOD B	**0.893**	0.107	0.429	0.107	0.429	**0.607**	−0.321	**1**												
CAT L	**0.679**	0.214	0.357	0.071	0.214	0.393	0.000	**0.821**	**1**											
CAT H	0.179	0.429	**0.643**	**0.821**	0.464	−0.250	0.429	0.179	0.286	**1**										
CAT K	**0.607**	0.286	0.250	0.214	0.143	−0.107	0.357	0.286	0.321	−0.214	**1**									
CAT B	0.286	0.250	**0.679**	**0.857**	0.500	−0.286	0.393	0.286	0.321	**0.964**	−0.143	**1**								
MDA L	0.396	0.360	0.342	0.450	0.000	−0.180	−0.288	−0.505	**0.577**	−0.324	−0.090	−0.270	**1**							
MDA H	0.342	0.234	0.126	0.036	0.487	**−0.631**	0.450	**−0.595**	**0.649**	−0.450	0.324	−0.414	0.191	**1**						
MDA K	0.393	**0.571**	0.179	0.429	0.107	−0.250	−0.071	−0.286	0.000	−0.286	−0.036	−0.214	**0.631**	−0.018	**1**					
MDA B	0.429	0.214	0.036	**0.750**	0.321	0.143	−0.429	−0.393	0.250	−0.429	−0.143	−0.464	**0.811**	−0.126	**0.750**	**1**				
GLU L	0.179	0.214	**0.786**	**0.750**	**0.571**	−0.393	0.429	0.143	0.250	**0.929**	−0.107	**0.964**	0.054	−0.378	0.000	−0.250	**1**			
GLU H	**0.571**	0.357	0.214	0.536	0.286	−0.143	0.143	**0.643**	0.536	0.357	0.214	**0.571**	0.288	−0.180	0.036	−0.500	0.500	**1**		
GLU K	**0.571**	0.214	0.000	0.464	0.036	0.143	0.000	**0.786**	**0.786**	0.429	0.036	**0.571**	0.505	−0.523	0.000	−0.464	0.464	**0.893**	**1**	
GLU B	0.500	0.143	0.357	0.536	0.107	0.071	−0.071	**0.643**	**0.571**	**0.643**	−0.107	**0.786**	0.144	**−0.631**	0.071	−0.250	**0.750**	**0.821**	**0.857**	**1**

*Bivariate Spermann rho correlation*. Bold values are: significant coefficient, *p* = 0.05 (bilateral test)

*Per* peroxidase, *SOD* superoxide dismustase, *CAT* catalase, *MDA* malonedialdehyde, *GLU* Glutathione, *L* liver, *H* heart, *K* kidney, *B* brain

## Organoprotective effects of the extracts

Oxidation of biomolecules is currently considered as the main molecular mechanism involved in the toxicity process that lead to cell death. The iron complex of the chelating agent nitrilotriacetic acid was used to induce lipid peroxidation. Our results show that Fe^3+^- NTA led to a significant increase of lipid peroxidation associated with SOD, catalase, and glutathione peroxidase activity depletion in all tissues assayed compared to the negative control. These results are in accordance with those of [51] which showed Fe^3+^-NTA to be nephrotoxic, hepatotoxic. Indeed, Fe^3+^- NTA may act through the generation of free radicals with simultaneous decrease in antioxidant defenses.

**Fig. 2:**
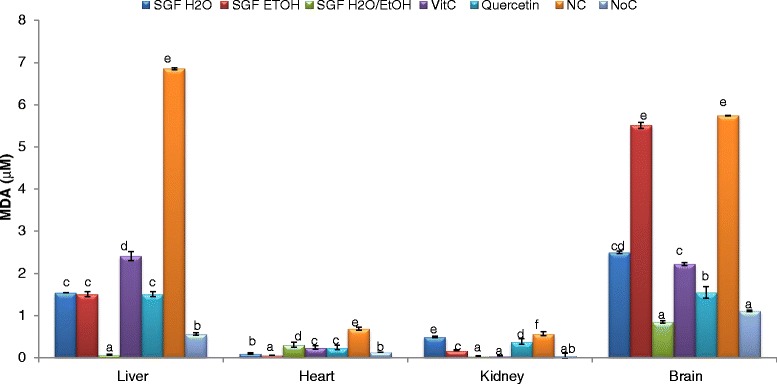
Lipid peroxidation inhibiting potential of barks extracts on different organ homogenates. The results are expressed as mean ± SD; For each organ homogenate, bars designated different letters are significantly different at *p* <0.05; SGFH_2_O/EtOH: Syzygium guineense macrocarpum (barks) aqueous-ethanol; SGFEtOH: syzygium guineense var macrocarpum (barks) ethanol; SGFH_2_O: syzygium guineense macrocarpum (barks) aqueous; Vit C: vitamin C; NC: negative control; NoC: Normal control

Lipid peroxidation is commonly quantified in research studies by measuring the accumulation of the by-products that result from this process. One of these by-products is malondialdehyde (MDA). The results of the protective effects of plant extracts against lipid peroxidation (Fig. 2) show that this property varied according to the plant extract and tissue homogenate. All the extracts lowered significantly (*p* < 0.05) the level of MDA when compared to the negative control. However, the aqueous- ethanolic extract exhibited the best protective activity by lowering 93, 13 and 6 fold respectively the MDA content in the liver, kidney and brain homogenates comparatively to the negative control. Conversely, in the heart homogenates the ethanol counterpart was the most active inhibitory effects of lipid peroxidation. These results highlight the beneficial effect of these plant extracts since lipid peroxidation is a major problem for food industry as well as for human health and it is associated to many diseases. Moreover, the inhibition of lipid peroxidation is confirmed by the restoration and potentialization of SOD, catalase, and peroxidase activities in plant-treated homogenates.

**Fig. 3:**
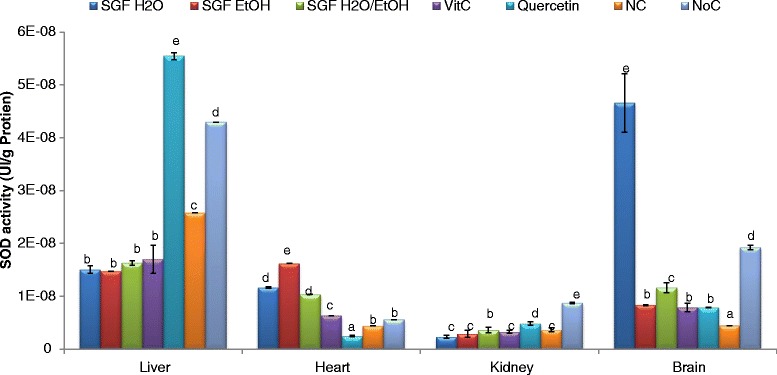
SOD protective effect of barks extracts on different organ homogenates. The results are expressed as mean ± SD; For each organ homogenate, bars designated different letters are significantly different at *p* <0.05; SGFH_2_O/EtOH: Syzygium guineense var macrocarpum (Barks) aqueous-ethanol; SGFEtOH: syzygium guineense var macrocarpum (barks) ethanol; SGFH_2_O: syzygium guineense var macrocarpum (barks) aqueous; Vit C: vitamin C; NC: negative control; NoC: Normal control

SODs are the first line of defense against oxidative stress among the highly sophisticated antioxidant system evolved by mammalian cells to cope with deleterious effects of ROS. The ability of the extracts to restore the SOD activity after oxidative stress induced are summarized in the Fig. 3. The ability of plant extracts to protect SODs is homogenate-dependent. In the liver, all the fractions exhibited a protective capacity (although not significant comparatively to the negative control). In the heart homogenate conversely, the ethanol extract was the most efficient among the tested extract whereas, the aqueous and aqueous-ethanolic counterparts restored more efficiently the enzyme activity in the brain and kidney homogenates respectively. The ability of the samples to protect the cells may be linked to their antioxidant content. Antioxidant molecules may act either as electron donors or hydrogen atom donors as pointed out by the DPPH^•^ and ABTS^+^ radicals scavenging potential or by modulating the antioxidant enzymes activities. Besides the aforementioned mechanisms, metal transition chelation is not to be excluded to this process. Indeed, numerous studies [52, 53] have shown that flavonoids present in the investigated samples in a significant higher amounts are efficient iron chelators. Furthermore, they can also act as hydrogen donors and superoxide anion quenchers.

**Fig. 4:**
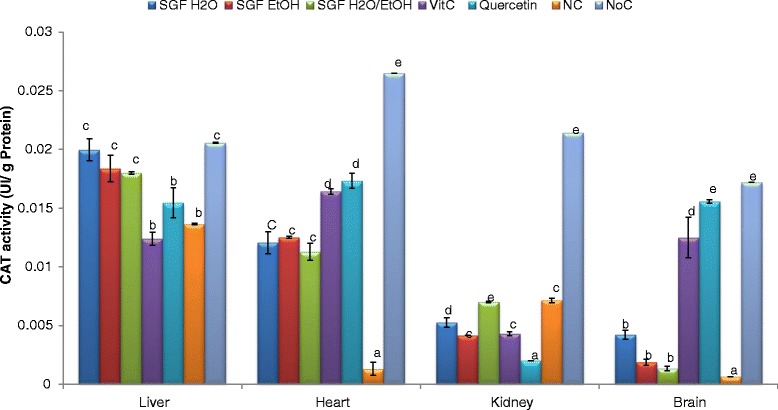
Catalase protective effect of Barks extracts on different organ homogenates. The results are expressed as mean ± SD; For each organ homogenate, bars designated different letters are significantly different at *p* <0.05; SGFH_2_O/EtOH: Syzygium guineense var macrocarpum (barks) aqueous-ethanol; SGFEtOH: Syzygium guineense var macrocarpum (barks) ethanol; SGFH_2_O: syzygium guineense var macrocarpum (barks) aqueous; Vit C: vitamin C; NC: negative control; NoC: Normal control

The ability of the plant extracts to potentiate the activity of catalase was also investigated since SODs are assisted by catalase in the conversion of hydrogen peroxide to water and oxygen. Results demonstrated that the exposure of liver, heart, brain and kidney homogenates to Fe^3+^-NTA reduces significantly the catalase activity in the negative control (homogenate treated only by the oxidant) while the treatment with all the sample extracts reversed significantly the depletion of the catalase activity (Fig. 4) in all the homogenates except the kidney where only the aqueous-ethanolic extract was significantly efficient. The aqueous extract exhibited the best protective effect of catalase both in the liver and brain homogenates whereas the aqueous-ethanolic and ethanol counterparts potentiate more the catalase activity in the kidney and heart homogenates respectively.

**Fig. 5:**
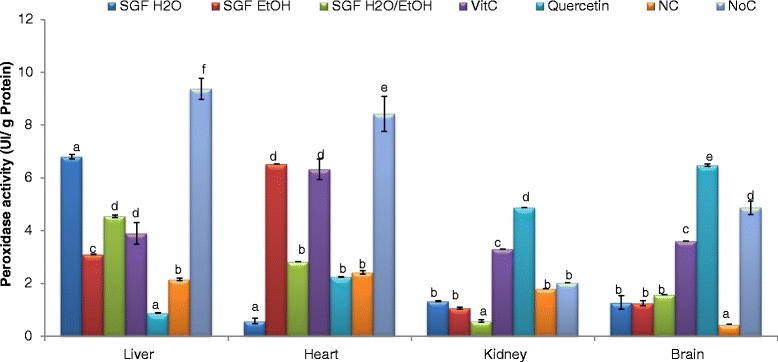
Peroxidase protective effect of Barks extracts on different organ homogenates. The results are expressed as mean ± SD; For each organ homogenate, bars affected with different letters are significantly different at *p* <0.05; SGF H_2_O/EtOH: Syzygium guineense var macrocarpum (barks) aqueous-ethanol; SGF EtOH: Syzygium guineense var macrocarpum (barks) ethanol; SGF H_2_O: Syzygium guineense var macrocarpum (barks) aqueous; Vit C: vitamin C; NC: negative control; NoC: Normal control

Removal of hydroperoxide which generates free radical is another way of preventing oxidation. Hydroperoxides can be reduced by enzymes such as glutathione peroxidase which cleared off the organism by eliminating organic hydroperoxide as well as hydrogen peroxide and peroxinitrites. The Fig. 5 displays the peroxidase protective potential of plant extracts against Fe^3+^ NTA-induced oxidative stress. From these results, aqueous extract was the most active sample both in the liver and kidney homogenates while the aqueous-ethanolic and ethanol extracts augmented more efficiently the activity of peroxidase in the heart and brain homogenates respectively. These results further confirmed the ability of these plant extract to protect biomolecules from oxidative damage since hydroperoxides decomposed rapidly to give many secondary products such as lipid free radicals which subsequently contribute to increase the oxidation of other molecules such as proteins, nucleic acids and other lipids leading to cancer, neurodegenerative pathologies and ageing [54].

**Fig. 6:**
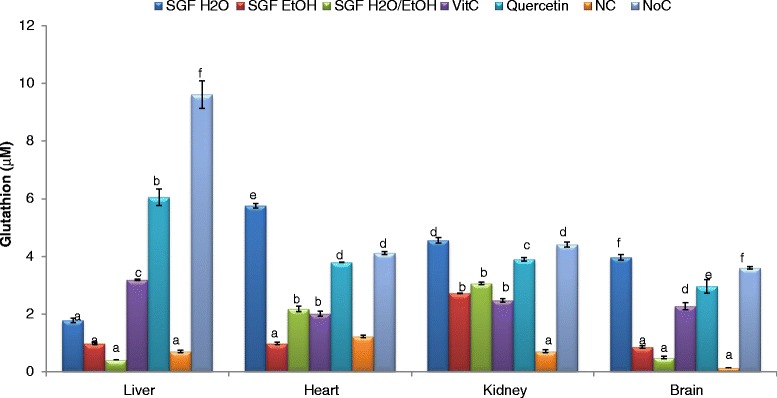
Glutathione protective effect of Barks extracts on different organ homogenates. The results are expressed as mean ± SD; For each organ homogenate, bars designated different letters are significantly different at *p* <0.05; SGFH_2_O/EtOH: Syzygium guineense var macrocarpum (barks) aqueous-ethanol; SGFEtOH: syzygium guineense var macrocarpum (barks) ethanol; SGFH_2_O: syzygium guineense var macrocarpum (barks) aqueous; Vit C: vitamin C; NC: negative control; NoC: Normal control

Reduced glutathione (GSH) is often considered as the body’s master antioxidant since it is used as a cofactor by multiple peroxidases, transhydrogenases, and glutathione S- transferases enzymes. Figure 6 exhibits the reduced glutathione levels in different organ homogenates. The aqueous extract showed the most beneficial effect (*p* < 0.05) compared to the other samples by repleting GSH levels in all the homogenates. Furthermore, this beneficial effect is higher than that of vitamin C and quercetin used as positive controls except in the liver homogenate. This result further confirms the ability of these plant extracts to protect vital organs from deleterious effects of ROS since GSH is largely known to minimize the lipid peroxidation of cellular membranes and other such targets that are known to occur with oxidative stress [55].

The Spearman correlation (Table 5A) studied was assessed to determine the degree of association between free radical scavenging efficacy and polyphenolic metabolites content in the one hand and between enzymatic antioxidant, lipid peroxidation markers of different homogenates (Table 5B). In general, results showed a positive and significant correlation between ABTS and FRAP (0.781; 0.05) on one hand, and ABTS and total phenol content (0.795; 0.05), flavonols (0.781; 0.05) and flavonoid (0.781; 0.05) on the other hand (Table 5A). Few significant and positive correlation were found between some markers of protective effects of the plant extracts investigated mainly SOD and peroxidase (Table 5B).

**Table 5:** Representation of the amounts of phenolic compounds in different bark extracts

Phenol standard		SGFH2O		SGFEtOH		SGFH2O/EtOH	
characteristics	Standard retention time (min)	A (mUA)	Conc (mg/g DW)	A (mUA)	Conc (mg/g DW)	A (mUA)	Conc (mg/g DW)
3.4-OH benzoic acid	19.10 ± 00						
Apigenin	33.49 ± 00	822207.6	186.91	26395.9	6.00	11129.0	2.53
Caffeic acid	25.67 ± 00						
Catechin	23.48 ± 00			26496.4	1925.87		
Eugenol	29.43 ± 00						
Gallic acid	14.38 ± 00						
O-coumaric acid	25.11 ± 00						
OH-tyrosol	21.91 ± 00			12969.4	1198.09		
P-coumaric acid	30.52 ± 00					21333.2	417.71
Quercetin	42.19 ± 00						
Rutin	29.45 ± 00			12728.4	1071.65		
Syringic acid	25.55 ± 00	53931.2	1337.02	16325.6	404.73	16427.9	407.26
Tyrosol	21.77 ± 00						
Vanillic acid	25.27 ± 00						

*A* area of the peak, *Conc* concentration of the standard in milligrams/grams of dried extract, *SGFH*_*2*_ *O/EtOH* syzygium guineense var macrocarpum (Barks) aqueous-ethanol, *SGFEtOH* Syzygium guineense var macrocarpum (barks) ethanol, *SGFH*_*2*_ *O* Syzygium guineense var macrocarpum (barks) aqueous

The plurality of methods used addition to the complexity of oxidative stress and variability of results from each method, have led us to perform a principal component analysis (PCA) to better determine the best extracts purely from a statistical point of view.

**Fig. 7:**
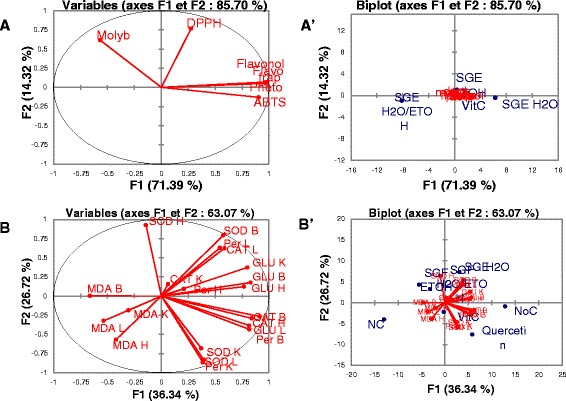
Degree of association between antioxidant capacity and free radical scavenging potential of different extracts. Principal Component Analysis test. **a** A’: Distribution of the tests, projection of the extracts and tests around the F1 and F2 axis; **b** B’: Distribution of the tests (from organs), projection of the extracts and tests (from organs) around the F1 and F2 axis. Molyb: Phosphomolybdenum test; Flavonols: Flavonol assay; Phetot: Polyphenol assay; Flavonoids: Flavonoid assay; ABTS: ABTS radical scavenging test; DPPH: DPPH radical scavenging test. SGFH_2_O/EtOH: Syzygium guineense macrocarpum aqueous-ethanol; SGFEtOH: syzygium guineense macrocarpum ethanol; SGFH_2_O: syzygium guineense macrocarpum aqueous; Vit C: vitamin C; NC: negative control; NoC: Normal control. Per: peroxidase; SOD: superoxide dismustase; CAT: catalase; MDA: malonedialdehyde; GLU: Glutathione; L: liver; H: heart; K: kidney; B: brain

From this analysis and the obtained results indicated that flavonols, total phenol content, flavonoids, and FRAP, are strongly correlated to the F_1_ axis with contribution percentages of 18.917, for flavonols, flavonoids and FRAP, and 18.914 for the total phenol content whereas phosphomolybdenum, and DPPH are closely loaded to F_2_ axis with 37.858 and 58.711 % contribution respectively (Fig. 7).

From the biplot (Fig. 7a’) the overall antioxidant effectiveness of plant extracts taking into account all the performed assays is as follows SGFEtOH/H_2_O > SGFH_2_O > SGFE**t**OH. Therefore the aqueous ethanolic extract of *S. guineense* var *macrocarpum* could be considered as the best extract regarding the antioxidant and protective effects.

## Conclusion

All the extracts (aqueous, ethanol and aqueous-ethanol) from *S. guineense* showed a concentration-dependent free radical. Similarly, the total antioxidant potential varied according to the solvent of extraction and the method used while the organo-protective properties also varied with regards to the homogenate. The HPLC characterization revealed the presence of six identified phenolic compounds belonging to different classes. However, syringic acid and apigenin were found in all the extracts. Although these plant extracts demonstrated important organo protective effect on all the tested homogenates by delaying or preventing lipid peroxidation and restoring enzymatic and non enzymatic markers activities, further studies need to be carried out to identify the active molecules and their mechanism of action.

## Abbreviations

ABTS: 2,2 -Azinobis(3-ethylbenzthiazoline)- 6-sulfonic acid
DPPH: 2,2-diphenyl-1-picrylhydrazyl 1,1-diphenyl-2- picrylhydrazyl radical
EDTA: Ethylene diamine tetra-acetic acid
FeCl_3_: Ferric chloride
FRAP: Ferric reducing antioxidant power assay
H_2_O_2_: Hydrogen peroxide
HCl: Hydrochloric acid
HPLC: High performance liquid chromatography
MDA: Malonaldialdehyde
NTA: Nitrilotriacetate
PBS: Phosphate buffer saline
PCA: Principal component analysis
ROS: Reactive oxygen species
SGF H_2_O/EtOH: *Syzygium guineense var macrocarpum* aqueous-ethanolic extract (barks)
SGFEtOH: *Syzygium guineense var macrocarpum* ethanolic extract (barks)
SGFH_2_O: *Syzygium guineense var macrocarpum* aqueous extract (barks)
SOD: Superoxide dismutase
TBA: Thiobarbituric acid
TCA: Trichloroacetic acid
UV rays: Ultraviolet rays
Vit C: Vitamin C
